# The role of MEK inhibition in pediatric low-grade gliomas

**DOI:** 10.3389/fonc.2024.1503894

**Published:** 2024-12-20

**Authors:** Shehryar R. Sheikh, Laura J. Klesse, Ross Mangum, Ashley Bui, Benjamin I. Siegel, Mohamed S. Abdelbaki, Neha J. Patel

**Affiliations:** ^1^ Department of Neurosurgery, Cleveland Clinic, Cleveland, OH, United States; ^2^ Department of Molecular Medicine, Case Western Reserve University, Cleveland, OH, United States; ^3^ Department of Pediatrics, Division of Hematology/Oncology, UT Southwestern Medical Center, Dallas, TX, United States; ^4^ Center for Cancer and Blood Disorders, Phoenix Children’s Hospital, Phoenix, AZ, United States; ^5^ Brain Tumor Institute, Children’s National Hospital, Washington, DC, United States; ^6^ Division of Neurology, Children’s National Hospital, Washington, DC, United States; ^7^ Division of Hematology and Oncology, Department of Pediatrics, School of Medicine, Washington University, St. Louis, MO, United States; ^8^ Department of Pediatric Hematology-Oncology and Blood and Marrow Transplant, Cleveland Clinic, Cleveland, OH, United States

**Keywords:** pediatric low grade glioma, PLGG, MEK, MAPK, targeted therapy, MEKi

## Abstract

Pediatric low-grade gliomas (pLGGs) are the most common brain tumors in children. Many patients with unresectable tumors experience recurrence or long-term sequelae from standard chemotherapeutics. This mini-review explores the emerging role of MEK inhibitors in the management of pLGGs, highlighting their potential to transform current treatment paradigms. We review the molecular basis for therapeutic MEK inhibition in the context of pLGG, provide an evidence base for the use of the major MEK inhibitors currently available in the market for pLGG, and review the challenges in the use of MEKi inhibitors in this population.

## 1 Introduction

Pediatric low-grade gliomas (pLGGs) are the most common brain tumors in children, constituting approximately 30% of all central nervous system (CNS) tumors in this population (1, 2). The majority of children with pLGG survive well into adulthood (3, 4); this realization has led to a gradual shift in our conceptualization of pLGG into a chronic disease paradigm wherein emphasis is placed on reducing long-term morbidity to optimize functional outcomes (5). Since the early 1990s, chemotherapy (particularly the combination of carboplatin and vincristine) has been the mainstay of treatment in United States (6). The 5-year progression-free survival with chemotherapy in patients with sporadic pLGG is around 40% and around 65-85% in patients with neurofibromatosis type 1 (NF-1) associated pLGG (7, 8). Though specific chemotherapy protocols vary across the globe, no regimen has shown consistently superior outcomes compared to these historical benchmarks.

Throughout the past decade, multiple landmark studies have provided novel insights into the molecular landscape of pLGGs. The understanding that this disease harbors fewer somatic driver alterations relative to other cancers has made molecularly targeted therapies a particularly exciting avenue. Specifically, the role of mitogen-activated protein kinase (MEK), a serine-threonine kinase in the classic RAS-MAPK signaling cascade, has become a keen area of focus for therapeutic inhibition. In this mini-review, we provide a primer on the role of MEK inhibition in pLGG. The text is organized into the following sections: 1) we recapitulate the key molecular biology mechanisms that underlie pLGG pathogenesis and thereby lay molecular rationale for MEK inhibition as a treatment paradigm, 2) we review the major MEK inhibitors available in the market and present the results of key clinical trials, and 3) we discuss key considerations in the contemporary clinical use of MEK inhibition in pLGG.

## 2 Role of MAPK/ERK in pLGG pathogenesis

**Figure 1:**
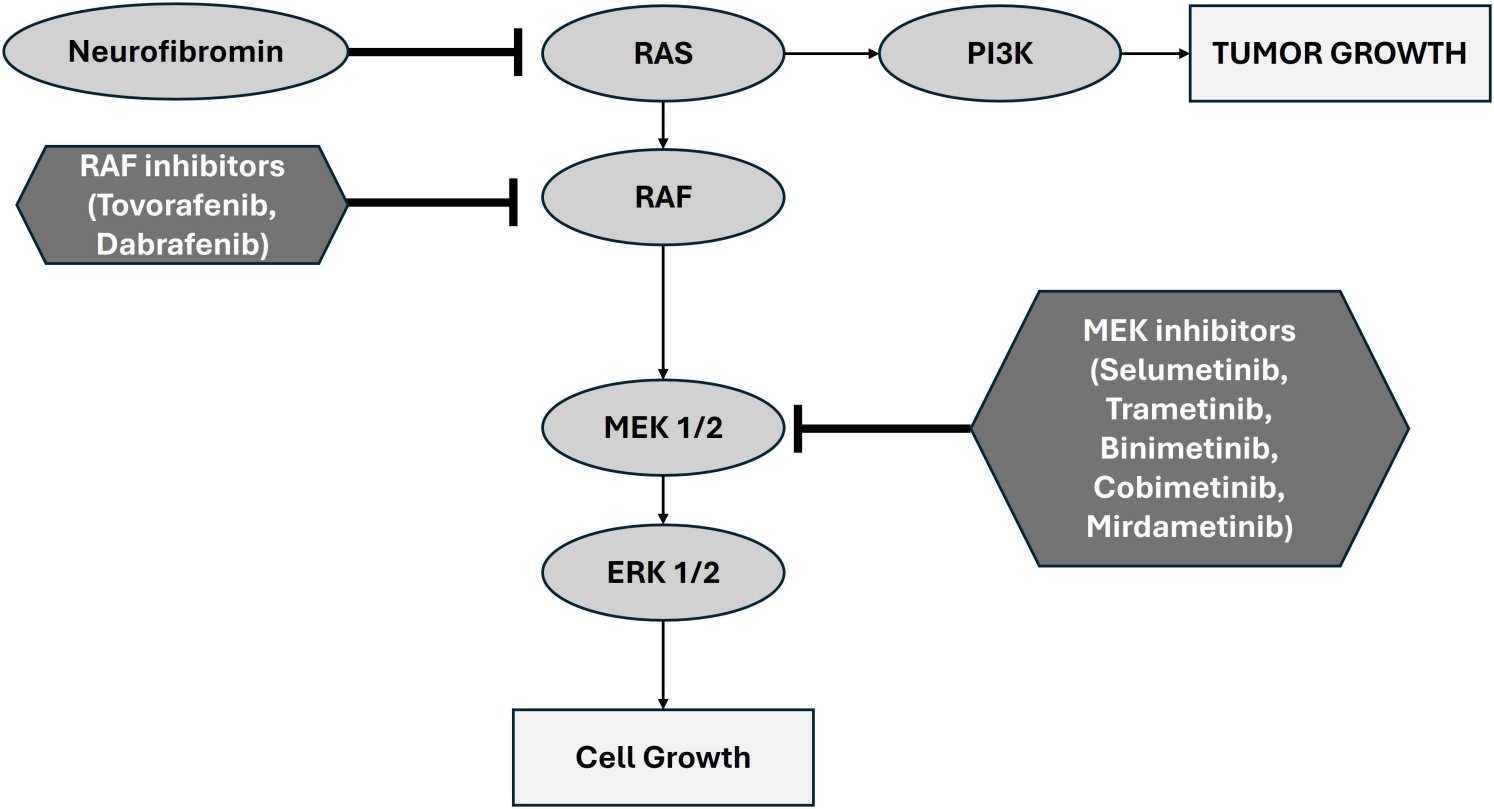
Schematic representation of the canonical MAPK/ERK pathway showing the role of MEK1/2 and the basis of therapeutic inhibition. Adapted from Zhao et al., 2014 (19).

Over the last decade, multiple large genomic studies have identified the key somatic driver events for pLGG (9–13). The most important discovery from these studies has been that the disease is driven primarily by alterations in the MAPK/ERK (mitogen-activated protein kinase/extra-cellular signal-regulated kinase) pathway (8). Canonically, MAPK/ERK is a critical signaling pathway regulating multiple cellular processes including growth, proliferation, differentiation, and survival. In physiological conditions, the pathway is initiated by an extracellular signal (e.g. growth factors, cytokines, hormones) binding to a receptor tyrosine kinase (RTK) which causes dimerization and autophosphorylation. The activated RTK then recruits and activates RAS, a GTPase, which in turn activates RAF, a protein kinase (including the notable isoform BRAF). RAF in turn phosphorylates and activates MEK, another kinase enzyme. MEK activates ERK which translocates into the nucleus where it phosphorylates transcription factors thereby altering gene expression. Relatedly, neurofibromin (a protein encoded by the NF1 gene) accelerates deactivation of RAS thus acting as a negative regulator of the pathway (**Figure 1**).

Structural variations in the *BRAF* gene are the most common somatic driver event for sporadic (non-NF1-related) pLGG, found in roughly 70% of cases (13, 14). These fusion events create abnormal gene products such as KIAA1549-BRAF which lead to the constitutive activation of the MAPK pathway. A smaller, but still significant, subset of pLGGs harbor single nucleotide polymorphisms in *BRAF* (most frequently *BRAF^V600E^*) which result in upregulated protein activity (15). One in five children with NF1 develop pLGG in the central nervous system, with approximately one third occurring in the optic pathway (16). Over 90% of pLGGs in NF1 patients harbor bi-allelic inactivation of NF1 without the BRAF mutations that are common in sporadic cases (17). A subset of NF1-assocaited pLGGs carry a higher risk of progression and treatment-resistance. This clinical heterogeneity is thought to be driven by acquisition of additional molecular alterations (18). For example, alterations in ATRX, CDK2A, and TP53, genes typically associated with high grade gliomas, are found infrequently in NF1-associated pLGGs and may portend more aggressive behavior.

### 2.1 Rationale for MEK inhibition

Given the central role of the MAPK pathway in pLGG pathogenesis, targeting MEK, a key kinase in this pathway, presents a rational therapeutic strategy. MEK inhibitors can potentially interrupt the aberrant signaling cascade driving pLGG pathogenesis, thereby halting tumor progression and inducing tumor regression in some cases. MEK inhibition is promising because of the potential for target specificity in comparison with classic chemotherapy (carboplatin and vincristine) raising the possibility of more limited toxicities and side effects. Furthermore, there is a key distinction between MEK1/2 and other protein targets within the MAPK/ERK pathway; MEK1/2 are kinases that activate ERK1 and ERK 2 but do not have any other known physiological substrates (19). In contrast, ERK1/2 catalyzes phosphorylation of countless cytoplasmic and nuclear targets for a diverse array of cellular functions. MEK1/2 thus serves as a relatively selective (and thus strategic) targetable bottleneck in tumorigenesis.

Though we will focus here on the role of MEK inhibition, it is worthwhile to note that in 2024 the FDA approved a highly selective type II RAF kinase inhibitor (Tovorafenib) pursuant to the results of a multicenter, open-label, single-arm trial in children with relapsed or refractory pLGG harboring an activating BRAF alteration (FIREFLY-1, NCT04775485) (20). The approval of Tovorafenib serves as an encouraging proof of concept supporting a focus on highly selective targeting of the RAS/RAF/MAPK pathway in pLGG. Relatedly, LOGGIC/FIREFELY-2 (NCT05566795) is currently enrolling patients and will serve as a Phase 3 trial investigating the efficacy of Tovorafenib monotherapy compared with chemotherapy in children with *newly diagnosed* LGG harboring an activating RAF mutation (21).

## 3 Major MEK inhibitors that have been clinically adopted

Multiple trials have experimented with the use of MEK inhibitors in different cancers that harbor mutation in the MEK pathway. Here, we review the available MEK inhibitors and the published clinical data on their role in pLGG (**Table 1**):

**Table 1:** Summary of major MEK inhibitors which have been trial in for pLGG. Adapted in part from de Blank et al. (34).

Agent	Supporting evidence in pLGG	Dosing in pLGG	Formulation	Common Grade 3/4 AEs	Half-Life	FDA Approval
Selumetinib	Phase II evidence, recurrent/refractory cases: pLGG with BRAF mutations: sustained PR in 36% NF1 associated pLGG: sustained PR in 40% (23)	25mg/m^2^ twice daily	Capsule	CK increase, maculopapular rash	5.3-7.2 hours	Approved for children with symptomatic, inoperable NF1 plexiform neurofibromas
Trametinib	Phase II evidence, relapsed/refractory cases: Overall response for pLGG with BRAF V600 mutations treated with combination trametinib and dabrafenib: 47% Median progression free survival was 20.1 months (compared to 7.4 months with chemotherapy) (28)	0.032mg/kg once daily for age <6 years and 0.025mg/kg once daily for age 6 years and above.	Tablet, oral solution	Hypertension, rash, pyrexia	4-5 days	Approved for children ≥1 year with newly diagnosed BRAF v600E mutant pLGG.
Binimetinib	Phase II evidence, recurrent/progressive cases: pLGG with BRAF fusion: overall response rate 50% pLGG associated with NF1: overall response rate 43% sporadic Plgg without BRAF fusion: overall response 69% (30)	32mg/m^2^/dose twice daily	Tablet, suspension (pharmacy prepared)	Rash, anemia, fatigue, dyspnea	3.5 hours	Approved in combination with encorafenib for BRAF-mutant melanoma
Cobimetinib	Phase I/II evidence, relapsed/refractory cases: pLGG with known/expected MAPK involvement, 9% had PR (31)	0.8mg/kg tablet, 1.0 mg/kg suspension	Tablet, suspension	Diarrhea, rash, fatigue	43.6 hours	Approved in combination with vemurafenib for metastatic melanoma
Mirdametinib	Phase I/II evidence, recurrent/progressive cases: pLGG with known MAPK pathway activation (except BRAF V600E), objective response in 63% (32)	3mg/m^2^/dose twice daily	Capsule, liquid	Thrombocytopenia, rash, alkaline phosphatase abnormality	8.6 hours	Not FDA approved

### 3.1 Selumetinib

Selumetinib is an orally available non-ATP-competitive small molecular inhibitor of MEK1/2. In 2017, a phase I trial of selumetinib in pediatric patients with recurrent or refractory pLGG was published by the Pediatric Brain Tumor Consortium (PBTC). The trial established a recommended phase 2 dose (RP2D) and reported a 2-year progression-free survival (PFS) at RP2D of 69% (SE 9.8%) (22). In a subsequent phase II trial in children with BRAF-aberrant or NF1-associated recurrent, refractory, or progressive LGG, imaging response rates were in the 30-40% range (23). Of the patients with BRAF aberrations (n=25), 36% achieved sustained partial response (PR) (median follow-up 36.4 months). Similarly, patients with NF1-associated tumors (n=25) achieved 40% sustained PR (medial follow-up 48.6 months). The study also reported on visual outcomes in 10 patients with optic pathway gliomas (all NF1-associated); 8 reported stable vision and 2 reported improvement. Elevated creatinine phosphokinase and maculopapular rash were the most frequent grade 3 or higher adverse events, both occurring in about 10% of patients. Notably, 36% of patients required a dose reduction due to toxicity, either grade 2 or grade 3. Although common, elevation of creatine phosphokinase was not symptomatic in most treated patients and the requirement for dose reduction based solely on this laboratory finding is an ongoing discussion in the treating community. The results of this phase II trial generated great excitement given that the tumor response with selumetinib therapy appeared comparable to that with chemotherapy but with significant advantages including oral administration without the need for central lines, no immunosuppression, less hair loss, and reduction in number of clinic visits (24). Results in a separate stratum of the same phase II trial that involved recurrent optic pathway or hypothalamic LGG in patients without NF1 (n=25) showed that 24% had PR, 56% had stable disease (SD), and 20% had disease progression (25). Visual acuity improved in 26% and was stable in the rest.

These results led to the current phase III, non-inferiority trials through the Children’s Oncology Group (COG) comparing selumetinib with standard-of-care chemotherapy (carboplatin and vincristine) for newly diagnosed pLGG. ACNS1821 (NCT03871257) is being conducted in children with NF1-associated pLGG, while ACNS1833 (NCT04166409) is being conducted in children with non-NF1 and non-BRAF^V600E^ mutant pLGG.

Though not in the context of pLGG, we note that compelling long term safety data for the use of selumetinib comes from the SPRINT trial (NCT01362803) conducted in the context of inoperable symptomatic plexiform neurofibromas (PN) in children with NF1. In the phase I study (n=24), investigators had reported only 1 grade 4 AE (asymptomatic elevated CPK). In the phase II study (n=50), three grade 4 AEs had been reported (CPK increase, hyperuricemia, and skin ulceration). In a five-year follow-up report after publication of phase II results, investigators reported that there had been no new or concerning safety signals in patients from the phase I or phase II studies (26).

### 3.2 Trametinib + Dabrafenib

Trametinib is a reversible, orally bioavailable, allosteric inhibitor of MEK1/2. In 2022, the FDA approved the combination of trametinib and dabrafenib (a BRAF inhibitor) in a tumor-agnostic fashion for the treatment of patients ≥ 6 years of age with unresectable/metastatic BRAF V600E-mutant solid tumors that have progressed after prior treatment and have no satisfactory alternative. As part of the supporting data for this approval, Bouffet et al. reported initial results from NCT02124772, a phase I/II study in children with relapsed/refractory BRAF V600-mutant pLGG treated with either trametinib monotherapy or in combination with dabrafenib (27). The PR rate was 15% in the monotherapy group (n=13) and 25% in the combination therapy group (n=36). Median PFS with combination therapy was 36.9 months, compared to 16.4 months with monotherapy. In the monotherapy group, common adverse events were paronychia (54%), diarrhea (46%) and dry skin (46%). Adverse events led to discontinuation in 54% of patients. In the combination group, the most common adverse events were pyrexia (50%) and dry skin (42%). Adverse events led to discontinuation of combination therapy in 22% of patients. A follow-up phase II trial, NCT02684058, studied the use of combination trametinib + dabrafenib (T+D) as first-line treatment for BRAF V600-mutant pLGG compared with chemotherapy (C+V) (28). Seventy-three children were treated with MAPK inhibition (T+D) and 37 with chemotherapy (C+V). The trial reported a higher overall response rate (ORR) in the group treated with MAPK inhibition (47% vs 11%, p< 0.001) and higher median PFS (20.1 months versus 7.4 months, p<0.001) when compared to the chemotherapy group. The most frequent adverse events in the T+D group were pyrexia (68%), headache (47%), and vomiting (34%), but there were fewer grade ≥3 adverse events in the T+D group and fewer discontinuations (4% versus 18%) as opposed to the carboplatin and vincristine group. Subsequent to the emergence of these data, the FDA approved D+T for children ≥1 year of age with newly diagnosed BRAF V600E mutant pLGG in March 2023. Given these data, the combination of trametinib and dabrafenib is now considered the standard of care for this select group of patients.

The utility of trametinib as a single agent in pLGGs without BRAFV600E alterations has not been well studied and currently only retrospective cohort data are available (29–31). Manoharan et al. reported on a retrospective cohort of patients treated with trametinib for progressive pLGGs and demonstrated a small percentage of PR and minor responses (MR) with most patients having SD on therapy (31).

Additional studies focused on trametinib monotherapy are currently underway, including PLGG-MEKTRIC, a large prospective trial comparing daily trametinib monotherapy with weekly vinblastine in children with non-NF1 associated pLGG and without BRAFV600E mutation in France (NCT05180825), and TRAM-01, a phase 2 open-label basket trial in Canada (NCT03363217), is testing trametinib as a single agent in pediatric patients with progressing/refractory glioma or plexiform neurofibroma with MAPK.ERK pathway activation.

### 3.3 Binimetinib

Binimetinib is an oral selective MEK 1/2 inhibitor currently FDA approved in the US for use in combination with BRAF inhibitor encorafenib in adult patients with unresectable BRAFV600 altered melanoma and non-small cell lung cancer. A multi-institutional phase II trial was conducted to evaluate the use of binimetinib in children with previously treated radiographically progressive pLGG (32). Radiographic response rates at 1 year were: 50% in pLGG with BRAF mutation (total n=28, 12 PR, 2 MR), 43% in NF1-associated pLGG (total n=21, 5 PR, 4 MR), and 69% in sporadic pLGG without BRAF mutation (total n = 29, 10 PR, 10 MR). The most common toxicities, as with other MEK inhibitors, were predominantly dermatologic and therapy had to be discontinued in 22% of patients, while 49% required a dose reduction due to toxicity.

### 3.4 Cobimetinib

Cobimetinib is an orally active, highly selective small molecule inhibitor of MEK1. iMATRIX-cobi (NCT02639546) was a multicenter phase I/II study investigating cobimetinib in pediatric patients with relapsed or refractory solid tumors with known/expected MAPK pathway involvement (33). Thirty-two patients with LGG were enrolled. Three patients (9%) had PR and 18 (56%) had SD (median follow-up 15 months). Eleven percent of patients (n=6) experienced adverse events requiring discontinuation from drug. It should be noted that 5 out of 6 of these patients experienced an ocular toxicity: one patient had grade 2 retinal detachment, 2 had chorioretinopathy (1 grade 2, 1 grade 4), one had grade 1 pigment epithelial detachment, and one had grade 1 serous retinal detachment. The higher incidence of ocular toxicities with this agent is notable given the interest in using MEKi for optic pathway gliomas wherein the tolerance for such adverse events is particularly low.

### 3.5 Mirdametinib

Mirdametinib is an oral small molecule allosteric inhibitor of MEK1/2. Though not yet available in the market, a New Drug Application (NDA) for the agent has recently been accepted by the FDA. SJ901 (NCT04923126) is a multi-arm phase I/II trial of mirdametinib in recurrent/progressive MEKi-naïve pLGG (excluding BRAFV600); results from phase I have recently been published (34). In 23 enrolled patients with median follow-up of 14.6 months, only one dose limiting toxicity (grade 3 thrombocytopenia) was seen. Twelve (63%) of the nineteen patients with measurable tumors achieved and objective response (OR) (of these, 1 major, 6 partial, and 5 minor). RP2D has been established (3mg/m^2^/dose BID) and phase II is ongoing.

## 4 Key considerations in clinical use

### 4.1 Patient selection

Determining appropriate selection of patients is perhaps the most challenging aspect of translating our burgeoning understanding of the molecular basis of pLGG into clinical practice. Not all pLGG – even with very similar genetic profiles - are likely to benefit equally from MEK inhibition and evidence-based decision-making paradigms are scant. Sigaud et al. have recently published results for a MAPKi sensitivity score (MSS) (35). Using pLGG transcriptomic data from large databases, they were able to show that computational models could predict the response of cell lines to drugs inhibiting different aspects of the MAPK pathway including MEKi. A key finding of their work was that accurate predictions relied not only on genetic information from the tumor cells but also the tumor immune microenvironment. Higher susceptibility scores correlated with higher immune cell infiltration (i.e. high expression in microglia compartment in single-cell RNA sequencing). This latter finding underscores the enormous complexity involved in predicting how a given patient will respond to MAPK inhibition based on molecular markers alone. Though not yet prospectively validated and thus not currently generalizable to routine clinical practice, the Sigaud method allows us to be optimistic about the development of computational tools that could be used in concert with the genetic profiling which is increasingly routine in the care of pLGG patients to make more personalized data-driven treatment recommendations.

### 4.2 Treatment duration and post-discontinuation re-growth

There is no consensus on the appropriate duration of treatment with MEK inhibitors for pLGG, though 2 years has become a common yardstick in clinical practice as this was the duration of most pLGG focused trials(though without a clear scientific rationale) (36). Use of MEK inhibitors is not limited to the pediatric population, however, and their use has been longstanding in adult tumors, particularly in melanoma. An increasingly common paradigm is to cease therapy after reaching prolonged CR. This notion is based on small cohort studies wherein most patients who achieved prolonged CR were monitored long term (8-36 months) without recurrence (37). It is challenging, however, to translate this principle into the care of pLGG patients treated with MEKi as CR is rare in our patients. Differences in tumor biology and behavior require adjustments to treatment strategy. Some pLGGs will remain stable for many years in a state of clinical senescence. Prolonged exposure to MEKi and the potential toxicities may not be warranted for this length of time and is likely not sustainable for most patients. Furthermore, the phenomenon of clinical senescence greatly complicates our ability to confidently ascertain whether tumor stability is related to therapeutic MEK inhibition as opposed to the natural history of the tumor.

A related concern in pLGG, not seen in most adult indications, is the rapid regrowth of disease soon after discontinuation. In the phase II selumetanib trial discussed earlier, 56% of patients with sporadic pLGG had disease progression with the majority occurring *after* therapy discontinuation. More strikingly still, in the NF1-associated pLGG stratum (n=25), only one patient progressed while on therapy while seven progressed after therapy discontinuation (23, 24). These studies have raised concern about the need for prolonged therapy in a subset of patients with recurrent, progressive disease and the possible merits of a slow wean of the drug. Such a ‘slow wean’ approach for MEKi has recently been endorsed in a consensus statement by Canadian experts (38).

### 4.3 Retreatment

Retreatment of patients with the same or alternative MEK inhibitors after progression with discontinuation of drug has become more accepted in clinical practice. There is some initial evidence supporting this type of re-retreatment. In the adult melanoma literature for instance, retreatment with combination MEK and BRAF inhibition is reasonable and is associated with at least temporary response (39). Specifically, in pLGG, preliminary evidence suggests that retreatment with selumetinib in patients who progress after initial therapy may be effective in restoring response or stability (40, 41). A key area of ongoing investigation is whether the addition of anti-resistance agents to MAPKi regimens may be a useful adjunct in retreatment paradigms. A current phase I/II trial through the PBTC (PBTC-055, NCT04201457) is testing addition of hydroxychloroquine to the existing combination of trametinib and dabrafenib (T + D + HCQ) in patients previously treated with a RAF or MEK inhibitor. The addition of HCQ is based on preclinical evidence showing that the agent can inhibit treatment induced autophagy which is a known pathway to treatment resistance in pediatric gliomas (42). Safety data for 18 evaluable subjects enrolled in the phase I component were comparable to prior MEK inhibitor trials; there were 2 dose-limiting toxicities, both grade 3 rash, and the highest dose level of HCQ was declared the RP2D (43).

### 4.4 Intermittent dosing

There has been substantial interest in the idea of using intermittent dosing (or ‘drug holidays’) as a way of mitigating treatment related toxicities while potentially decreasing resistance to MAPK inhibition. Again, population specific evidence for pLGG is lacking but reports from adult studies may be at least partially informative. A randomized phase II trial in adult melanoma patients investigated continuous versus intermittent dosing of combination trametinib and dabrafenib in metastatic or unresectable BRAF^V600E^- mutant melanoma (44). There were no significant differences in terms of toxicity or OS however patients in the continuous dosing arm had improved PFS. In another trial, continuous dosing of combination cobimetinib and vemurafenib (a Type 1 BRAF inhibitor) was compared with intermittent dosing in patients with advanced BRAF-mutant melanoma (45). Again, patients in the intermittent dosing arm did not have improved PFS or substantially reduced toxicities. Though these adult melanoma studies are imperfect proxies for the pLGG context given the significant difference in the disease pathophysiology and natural history, they do provide evidence for the notion that an intermittent dosing approach should be viewed with caution at this time. Clinical trials testing the specific question of whether intermittent dosing may be beneficial for pediatric glioma patients are underway; NCT03326388 (INSPECT) is a phase I/II study in children with NF1-associated tumors (inoperable plexiform neurofibromas and recurrent optic pathway gliomas) being treated with intermittent dosing of selumetinib. Notably, the recently published ReNeu trial found that children with NF-1 associated inoperable symptomatic plexiform neurofibromas treated with intermittent dosing of Mirdametinib (3 weeks on/1 week off) met the primary efficacy endpoint, with ORR of 52% (46).

### 4.5 Potential impact on fertility

Given the long term survival of most pLGG patients, there has been a sustained interest in establishing the impact of targeted therapies (including MEKi) on fertility, but compelling data in human subjects remain scant. Concerns about the potential toxicity to fertility come from animal studies. In female rats, cobimetinib exposure increases apoptosis and necrosis of cells in the corpus luteum and vaginal epithelium while also causing testicular degeneration in male dogs (47). Trametinib has also been associated with reduced corpus leuteum cells in female rats, but no observed effect in male reproductive tissues (47). To our knowledge, there are currently no data in humans to better inform the long-term risks to fertility associated with MEKi use (48).

## 5 Discussion

While chemotherapeutics have been the mainstay for the treatment of pLGG since the early 1990s, our understanding of the molecular drivers of the disease has deepened markedly in the last decade leading to the widespread appreciation of the role of targeted therapies. Unlike other cancers, pLGG is driven by relatively few somatic driver mutations primarily impacting the MAPK/ERK pathway. MEK inhibition has now been evaluated in multiple phase I/II trials in both sporadic and NF1 associated pLGG and early results suggest that for appropriately selected patients outcomes are likely to be as good as (if not superior to) those with conventional chemotherapies. The emergence of the MAPK pathway alterations and the activity of targeted agents has made molecular characterization of the tumor a required aspect of diagnosis and management. Several questions remain, however, in the continued use of this targeted therapy. Identification of the patients most likely to benefit, the durability of therapy, the length of optimal therapy, and the process for therapy discontinuation remain clinical challenges. Much work is necessary to resolve these challenges before the treatment paradigm can shift to one wherein molecular targeted therapies are the standard treatment. Combination of MEKi with cytotoxic chemotherapy or combination of MEKi with other targeted therapies such as PD1 inhibition may be exciting future avenues that are yet to be explored.
